# Effects of miRNA-140 on the Growth and Clinical Prognosis of SMMC-7721 Hepatocellular Carcinoma Cell Line

**DOI:** 10.1155/2021/6638915

**Published:** 2021-02-05

**Authors:** Cun-qing Kong, Xing-cai Chen, Guan-hua Qiu, Jing-chen Liang, Duo Wang, Xin-yu Liu, Jun-jie Liu, Yao-qi Han, Xiao-hui Fan

**Affiliations:** ^1^Department of Microbiology, The School of Preclinical Medicine, Guangxi Medical University, Nanning, Guangxi 530021, China; ^2^Department of Anatomy, Guangxi Medical University, 22 Shuangyong Road, Nanning, Guangxi 530021, China; ^3^Department of Ultrasound, Affiliated Tumor Hospital of Guangxi Medical University, Nanning, Guangxi 530021, China; ^4^Department of Hepatobiliary Surgery, Affiliated Tumor Hospital of Guangxi Medical University, Nanning, Guangxi 530021, China

## Abstract

**Background:**

A growing number of studies have suggested that microRNAs exert an essential role in the development and occurrence of multiple tumours and act as crucial regulators in various biological processes. However, the expression and function of miRNA-140 in hepatocellular carcinoma (HCC) cells are not yet adequately identified and manifested.

**Methods:**

The expression of miRNA-140 was determined in HCC tissues and adjacent nontumour tissues by quantitative real-time polymerase chain reaction (qRT-PCR). Kaplan–Meier survival analysis and Cox regression analysis were performed to explore the correlation between miRNA-140 expression level and the survival rate of patients with HCC. Additionally, overexpression experiments were conducted to investigate the biological role of miRNA-140 in HCC cells. Bioinformatics was used to predict the related target genes and pathways of miRNA-140.

**Results:**

QRT-PCR results signified that the expression level of miRNA-140 in HCC was lower than that of adjacent normal tissues (*P* < 0.0001). Compared with the control group, the SMMC-7721 HCC cells in the miRNA-140 mimic group had a decrease in proliferation, migration, and invasion (*P* < 0.05), whereas those in the miRNA-140 inhibitor group had an increase in proliferation, migration, and invasion (*P* < 0.05). Cell cycle arrest occurred in the G0/1 phase. Prognosis analysis showed that the expression level of miRNA-140 was not related to the prognosis of HCC. Furthermore, the Kaplan–Meier test revealed that patients with lower miRNA-140 expression levels in liver cancer tissue had significantly shorter disease-free survival (DFS, *P* = 0.004) and overall survival (OS) times (*P* = 0.010) after hepatectomy. Cox regression analysis further indicated that miRNA-140 was an independent risk factor that may affect the DFS (*P* = 0.004) and OS times (*P* = 0.014) of patients after hepatectomy. Our results suggested that miRNA-140 might be a crucial regulator involved in the HCC progression and is thus considered a potential prognostic biomarker and therapeutic target for HCC.

## 1. Background

Hepatocellular carcinoma (HCC) is one of the most common malignant tumours. HCC ranks second amongst the causes of death in patients with cancer because of its high morbidity, high mortality, and poor prognosis [1, 2]. HCC is the third deadliest cancer with over 600,000 deaths per year worldwide [3, 4]. Despite the availability of new treatment methods, the overall survival (OS) rate of patients with HCC still remains fairly low; the 5-year survival rate becomes higher than 50% after resection for early-stage HCC [5]. Therefore, we need to develop a new therapy to effectively interfere with disease progression in patients with HCC. In this regard, the epigenetic changes in microRNA (miRNA) and its target gene expression may provide new tools and opportunities for HCC research.

miRNA is an endogenous, evolutionarily conserved, single-stranded, and noncoding RNA [6]. Its main role is the regulation of the expression of target genes in its 3′-untranslated region (UTR). In addition, miRNAs also participate in physiological processes, such as growth, metabolism, and apoptosis at the transcription level, by degrading or inhibiting gene translation [7, 8]. miRNAs play a crucial role in a variety of biological processes, including cell differentiation, proliferation, migration, invasion, apoptosis, and tumour metastasis, and are thus considered new biomarkers for tumour diagnosis and treatment [9, 10]. miRNAs could regulate hundreds of genes to play a functional role in human cancers [11]. Many scholars have investigated the role of miR-140 in tumour formation in various cancers, such as glioma [12], cervical cancer [13], gastric cancer [14], breast cancer [15], lung cancer [16], and osteosarcoma [17]. Some scholars have also studied the effect of miRNA-140 on liver cancer. For example, Zhang found that miR-140-3p suppresses the MAPK signalling pathway by targeting GRN and therefore inhibits epithelial-mesenchymal transition, invasion, and metastasis in HCC [18]. Yang et al. revealed that miR-140-5p is downregulated in HCC and affects HCC growth and metastasis by targeting fibroblast growth factor 9 (FGF9) and TGF-*β* receptor 1 in HCC [19].

miRNAs are abnormally expressed in HCC and participate in the growth, development, and metastasis of HCC by acting as oncogenes or tumour suppressors [20]. In the present study, our purpose was to observe the difference in the expression of miRNA-140 in HCC tissues and adjacent normal tissues and explore the effect of miRNA-140 on the clinical indicators and prognosis of HCC. In addition, we explored the role of miRNA-140 in HCC cells through cell function study.

## 2. Methods and Materials

### 2.1. Clinical Specimens

Eighty specimens of cancer and adjacent normal tissues were collected from the Department of Hepatobiliary Surgery of the Affiliated Tumour Hospital of Guangxi Medical University. The 80 patients met the following conditions: underwent radical hepatectomy, had pathologically diagnosed HCC, had no other treatments before surgery, had no other tumours, and had complete clinical and follow-up data. The corresponding adjacent normal liver tissues were collected more than 2 cm away from the tumour boundary, and normality was confirmed by pathological examination. The tissue samples were surgically removed from the patients with HCC, quickly placed into cryopreservation tubes with RNA protection solution, labelled, placed into a liquid nitrogen tank, and later transferred to a −80°C laboratory freezer for further preservation.

The HCC tissues and corresponding adjacent normal liver tissues of 198 patients with HCC were collected during hepatectomy from January 2014 to December 2016 in the Hepatobiliary Surgery Department of Guangxi Medical University Affiliated Tumour Hospital. The patients were followed up every 3 months within 1 year by outpatient reexamination and in the long-term by telephone. The follow-up deadline was September 2019. The study protocol was approved by the Hospital Ethics Committee. All patients signed the relevant informed consent form. The corresponding adjacent normal liver tissues were collected more than 2 cm away from the tumour boundary, and normality was confirmed by pathological examination. The tissues were stored at −80°C.

The clinical data of the patients with HCC, including data on their biological sex, age, smoking and drinking history, family history, body mass index (BMI), alpha-fetoprotein (AFP) value, liver cirrhosis, number of tumours, metastasis, Barcelona clinic liver cancer (BCLC) stage, and portal vein tumour thrombus (PVTT) were collected during hepatectomy from January 2014 to December 2016 in the Hepatobiliary Surgery Department of Guangxi Medical University Affiliated Tumour Hospital. The study was approved by the ethics committee of the Affiliated Tumour Hospital of Guangxi Medical University, and all patients signed the relevant informed consent forms.

### 2.2. Main Reagents

The reagents include RNA protection solution (Guangzhou Jianlun Science and Technology Biology Co., Ltd.); TRIzol total RNA extraction reagent (Adelair, China); isopropanol, ethanol, and chloroform (Tianjin Chemical Reagent Company); miRNA cDNA kit and miRNA PCR kit (Beijing Tiangen Co.); phosphate-buffered saline (PBS) powder, penicillin double-antibody, EDTA R-trypsin digestive fluid and EDTA-free protease digestive fluid (Solabo Company of China); Dulbecco's modified Eagle's media (DMEM), high-glucose medium, foetal bovine serum (FBS; Gibco Company, USA); Opti-MEM serum-free medium (Semefei Company); Hsa-miRNA-140 mimic, mimic negative control (NC), Hsa-miRNA-140 inhibitor, and inhibitor NC (Tiangen Biotech Co., Ltd., Beijing); Lipo6000 transfection reagent (China Biyuntian Biotechnology Co., Ltd.); cell cycle detection kit and annexin V–fluorescein isothiocyanate (FITC) cell apoptosis detection kit (China Bebo Biotechnology Co., Ltd.); transwell chamber (Costar, USA); and Matrigel-coated membrane (BD, USA).

### 2.3. Total RNA Extraction and Reverse Transcription Quantitative Real-Time Polymerase Chain Reaction (qRT-PCR)

The specific method for total RNA extraction is as follows. SMMC-7721 cells were extracted based on the instructions of the Tiangen total RNA extraction kit. The concentration and purity of the extracted RNA were detected using a UV spectrophotometer. The RNA was reverse transcribed into cDNA using Takara's reverse transcription kit, and the qRT-PCR results were later analysed by the 2^−*ΔΔ*Ct^ method using U6 as the internal reference.

### 2.4. Cell Line and Transfection

Human hepatoma cell line SMMC-7721 was purchased from the cell bank of Shanghai Life Science Research Institute. The cells were maintained in DMEM supplemented with 10% FBS, 100 U/mL penicillin, and 100 *μ*g/mL streptomycin and cultured in a humidified incubator with 5% CO_2_ at 37°C. The cells were transfected in a 6-well plate. According to the relevant reagent specifications, miRNA-140 mimic, mimic NC, miRNA-140 inhibitor, and inhibitor NC, as well as Lipo6000, were separately added into the pretransfection pores of a 6-pore plate. The expression of miRNA-140 in SMMC-7721 cells was subsequently detected by qRT-PCR after 48 h of transfection.

### 2.5. Cell Proliferation Assay

SMMC-7721 cells were inoculated into a 96-well plate (5 × 10^4^ per 100 *μ*L/well) after 48 h of transfection and then at 0, 24, 48, 72, and 96 h after inoculation. The original medium was discarded, and 120 *μ*L of medium containing 3-(4,5-dimethylthiazol-2-yl)-2, 5-diphenyltetrazolium bromide (MTT; serum − free medium to MTT = 5 : 1 preparation) was added into each well. The culture process was subsequently terminated after 4 h at 37°C in 5% CO_2_, and the in-hole medium was extracted with careful deliberation. In addition, 150 *μ*L of dimethyl sulfoxide was later added into each well. The viability of HCC cells was measured by measuring the optical density of the cells at 490 nm wavelength.

### 2.6. Colony Formation Assay

After 48 h of transfection, the cells were digested with membrane protease, centrifuged, added with complete medium, and serially diluted to prepare a single-cell suspension. Approximately 500 cells were added to each well of a 6-well plate to increase the volume of the entire medium to 2 mL. Three pores were seeded and assigned for the transfected cells. The 6-well plate was stored in a constant temperature incubator for 2–3 weeks. The culture medium was changed every 3 days until the cell clones became visible to the naked eye prior to the termination of the culture process. The cells were then fixed with 75% ethanol for 20 min, stained with 0.1% crystal violet for 30 min, rinsed with PBS, dried, and photographed.

### 2.7. Wound Healing Assay

Logarithmic cells (1 × 10^5^/L) were inoculated into each pore of the 12-pore plate and then transfected with miRNA-140 mimic, mimic NC, miRNA-140 inhibitor, and inhibitor NC. A set of 200-mL pipette tips, perpendicular to each hole above the surface of HCC cells, were labelled 48 h later. The floating cells were further rinsed with PBS and replaced with DMEM. Photos were captured at 0 and 24 h after scratches.

### 2.8. Migration and Invasion Assays

According to the experimental protocol, the Transwell cell was used to measure the migration or invasion ability of HCC cells. The polycarbonate film of the Transwell cell was precoated with or without Matrigel glue, placed in a 24-well culture plate, and inoculated with 5 × 10^4^ and 1 × 10^5^ cells. Then, the Transwell cell was added with DMEM without FBS to 200 *μ*L. DMEM with 10% FBS (500 *μ*L) was added to the lower chamber. The chamber was removed after 18–24 h. The cells that did not penetrate the membrane inside the chamber were wiped out. The remaining cells were fixed with methanol and stained with 0.1% crystal violet solution for 30 min. The number of transmembrane cells in five fields was counted under a microscope.

### 2.9. Determination of Cell Cycle by Flow Cytometry

A total of 5 × 10^5^ cells were collected and centrifuged at 1,200 rpm for 5 min prior to the removal of the supernatant. Next, 1 mL of precooled 75% ethanol was placed into each tube. All the collected cells were again suspended, fixed for more than 2.5 h at 4°C without light, and centrifuged for 5 min at 1,200 rpm. The relevant supernatant was discarded. The cells were cleansed with 1 mL of PBS and centrifuged. The supernatant was discarded. Finally, each sample was stained with 500 *μ*L of propidium iodide (PI)/RNase dye and incubated for 15 min. The whole incubation process was carried out at room temperature and protected from light. The entire process was completed within 0.5 h.

### 2.10. Cell Apoptosis

Cell apoptosis was detected by Annexin V–FITC/PI double staining. Cells (10^5^) were centrifuged at 1,200 rpm for 5 minutes, and the supernatant was removed. Each tube was added with 50 *μ*L 1× binding buffer. The cells were resuspended and added with dye according to the following groups: the negative tube was not added with dye, and the sample tube was added with 5 *μ*L of annexin V–FITC and 10 *μ*L of PI. The solutions were shaken gently, incubated at room temperature (25°C) in the dark for 15 min, and added with 200 *μ*L of 1× binding buffer. Cell apoptosis was determined using a computer software within 1 h.

### 2.11. Predictive Target Gene and GO Annotation and KEGG Pathway Enrichment Analysis

The target genes of miRNA140 were predicted by miRDB (http://www.mirdb.org/index.html), miRTarBase (http://mirtarbase.cuhk.edu.cn/php/index.php), miRWalk (http://mirwalk.umm.uni-heidelberg.de/), and TargetScan (http://www.targetscan.org/vert_72/). Gene Ontology (GO; http://www.geneontology.org) functional annotation and Kyoto Encyclopaedia of Genes and Genomes (KEGG; http://genome.ad.jp/kegg/) pathway enrichment analysis were performed on miRNA-140 target genes.

### 2.12. Statistical Analysis

The SPSS 22.0 software (Chicago, Illinois, USA) was used for statistical analysis, and GraphPad Prism 8 Project software (La Jolla, California, USA) was used for drawing. The measurement data in the experiment are expressed as mean ± standard deviation. Student's *t*-test was conducted to evaluate the miRNA-140 level between human HCC tissues and adjacent normal tissues. Qualitative variables were compared via *χ*^2^ tests. Kaplan–Meier survival analysis and Cox regression analysis were performed to compare the correlation between the miRNA-140 expression level and patient OS and disease-free survival (DFS) rates. Two-way *P* < 0.05 indicates a statistical difference.

## 3. Results

### 3.1. Downregulation of miRNA-140 Expression in Patients with HCC

**Figure 1:**
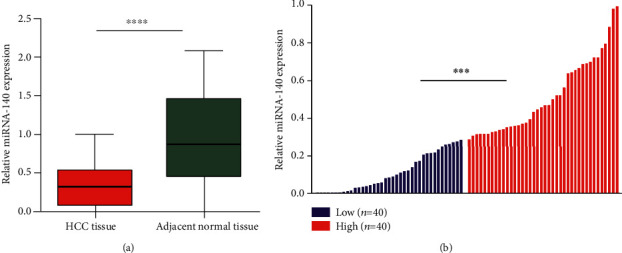
Relative expression levels of miRNA-140. (a) miRNA-140 expression in 80 paired HCC tissues and adjacent normal tissues. (b) Patients were divided into groups based on levels higher or lower than the median value in HCC tissues. ^∗∗∗∗^*P* < 0.0001; ^∗∗∗^*P* < 0.001.

QRT-PCR was used to determine the expression of miRNA-140 in 80 HCC tissues and adjacent normal tissues. The subsequent results signified that the expression level of miRNA-140 in adjacent normal tissues was significantly higher than that in the corresponding HCC tissues (*P* < 0.0001; Figure 1(a)). The expression level of miRNA-140 in HCC tissues was divided into high and low groups according to the median, and the correlation between miRNA-140 and clinicopathological characteristics was observed (*P* < 0.0001; Figure 1(b)).

### 3.2. Correlations between the Aberrant Expression of miRNA-140 and the Clinical Pathological Features of HCC

The results of the *χ*^2^ analysis indicated that the miRNA-140 expression was significantly associated with BCLC stage (*P* = 0.036) but not with other factors, such as biological sex, age, drinking history, family history, BMI, AFP, liver cirrhosis, number of tumours, and PVTT (Table 1).

### 3.3. Association between the miRNA-140 Expression and Prognosis of Patients with HCC

**Figure 2:**
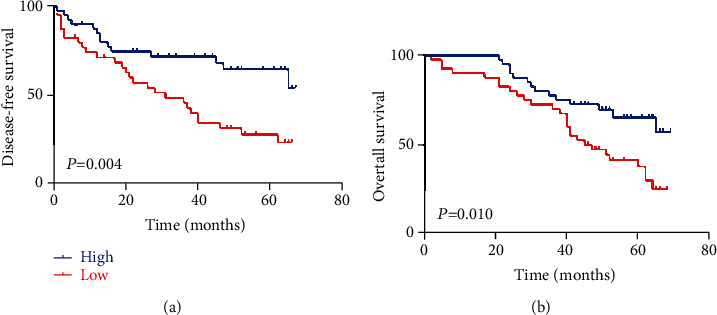
Kaplan-Meier analysis was used to evaluate the role of miRNA-140 in the prognosis of HCC patients. (a) Disease-free survival. (b) Overall survival.

The OS and DFS of the two groups were compared according to miRNA-140 levels. The relevant Kaplan–Meier curves of the low and high expression groups were generated, and both groups were subjected to a log-rank test. The subsequent results indicated that DFS was poorer in patients with low miRNA-140 expression than in patients with high miRNA-140 expression (23.21% and 53.86%, respectively, *P* = 0.004; Figure 2(a)). Patients with low miRNA-140 expression had poorer OS than those with high miRNA-140 expression (25.12% and 57.09%, respectively, *P* = 0.010; Figure 2(b)). Therefore, the prognosis of patients with low miRNA-140 expression after hepatectomy was remarkably worse.

### 3.4. Multivariate Regression Analyses of OS and DFS

All relevant indexes were contained in the multivariate Cox regression analysis (Table 2). The results pinpointed that the following independent risk factors affected the DFS of patients with HCC: low miRNA-140 expression, biological sex (female), age (<60 years), and number of tumours (≥3). The substantial independent risk factors that affect OS time include low miRNA-140 expression, biological sex (female), and age (<60 years). The results of the study found that the prognosis of the high miRNA-140 expression group may be better than that of the low expression group.

### 3.5. miRNA-140 Overexpression Inhibited HCC Cell Proliferation

**Figure 3:**
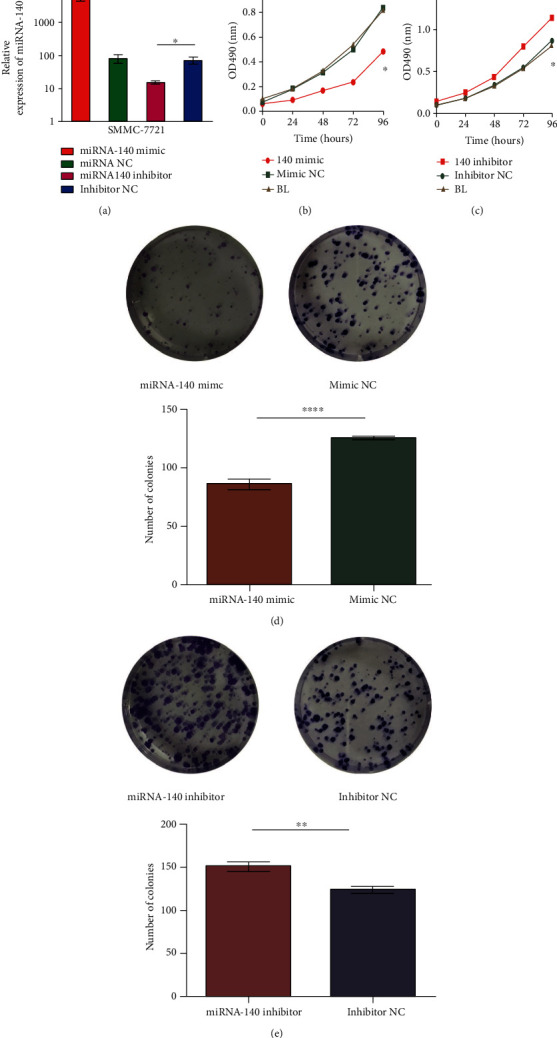
Overexpression of miRNA-140 inhibited HCC cell proliferation. Expression of miRNA-140 in SMMC-7721 cells after transfection. (a) Effect of miRNA-140 on the proliferation curve of SMMC-7721 cells. (b) miRNA-140 mimic and mimic NC. (c) miRNA-140 inhibitor and inhibitor NC. Changes in the number of clones formed in SMMC-7721 cells after upregulation and downregulation of miRNA-140. (d) miRNA-140 mimic and mimic NC. (e) miRNA-140 inhibitor and inhibitor NC. ^∗∗∗∗^, ^∗∗^, and ^∗^ represent *P* < 0.0001, *P* < 0.005, and *P* < 0.05, respectively.

We used qRT-PCR to verify the transfection level of miRNA-140 to examine whether miRNA-140 mimic and miRNA-140 inhibitor were transiently transferred into HCC cells. The relative expression level of miRNA-140 was higher in SMMC-7721 cells transfected with miRNA-140 mimic, whereas the relative expression level of miRNA-140 was lower in SMMC-7721 cells transfected with mimic NC (*P* < 0.0001) or miRNA-140 inhibitor than those transfected with inhibitor NC (*P* = 0.0261; Figure 3(a)). We explored the effect of miRNA-140 on the proliferation of SMMC-7721 cells. The results indicated that miRNA-140 mimic markedly inhibited the proliferation of SMMC-7721 cells compared with mimic NC and blank group (BL) (*P* < 0.05) and that the miRNA-140 inhibitor markedly promoted the proliferation of SMMC-7721 cells compared with the inhibitor NC and BL (*P* < 0.05; Figures 3(b) and 3(c)). No significant difference was observed amongst BL, mimic NC, and inhibitor NC (*P* > 0.05; Figures 3(b) and 3(c)). miRNA-140 overexpression inhibited HCC cell proliferation. Similarly, we found in the colony formation assay that the number of cell colonies in the miRNA-140 mimic NC group was higher than that in the miRNA-140 mimic group (*P* < 0.0001; Figure 3(d)), and the number of HCC cell colonies in the miRNA-140 inhibitor group was higher than that in the inhibitor NC group (*P* = 0.0015; Figure 3(e)). Therefore, the high expression of miRNA-140 inhibited the formation of cell colonies.

### 3.6. miRNA-140 Overexpression Inhibited HCC Cell Migration and Invasion

**Figure 4:**
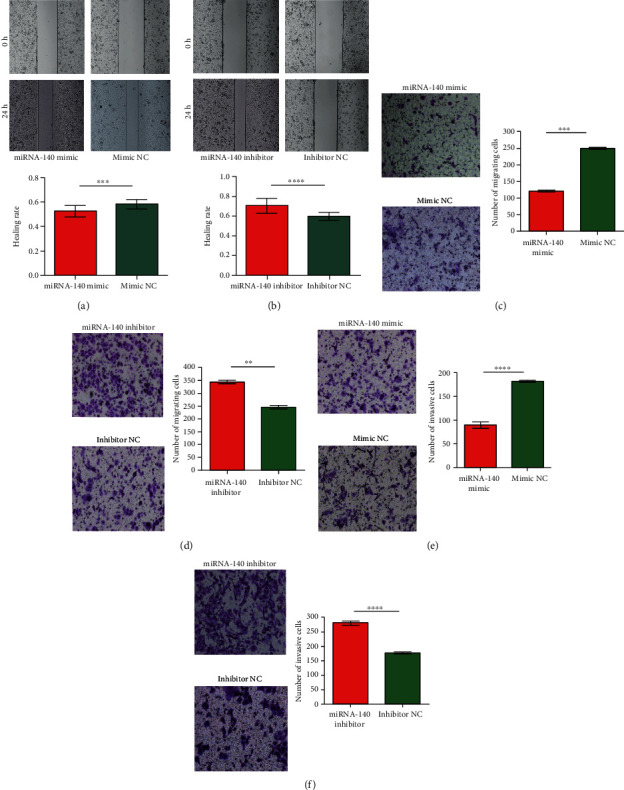
Overexpression of miRNA-140 inhibited the migration and invasion of HCC cells in vitro. The SMMC-7721 migration potential of the miRNA-140-transfected group and control group was detected by wound healing. (a) miRNA-140 mimic and mimic NC, (b) miRNA-140 inhibitor and inhibitor NC. Effect of miRNA-140 on the migration of SMMC-7721 cells. (c) miRNA-140 mimic and mimic NC. (d) miRNA-140 inhibitor and inhibitor NC. The effect of miRNA-140 on the invasiveness of SMMC-7721 cells. (e) miRNA-140 mimic and mimic NC. (f) miRNA-140 inhibitor and inhibitor NC. ^∗∗∗∗^, ^∗∗∗^, and ^∗∗^ represent *P* < 0.0001, *P* < 0.001, and *P* < 0.005, respectively.

Wound healing experiments and transwell assays were conducted to observe the effects of miRNA-140 on the migration and invasion of HCC cells. Cell wound healing experiments revealed that the rate of wound healing in the miRNA-140 mimic group was lower than that in the mimic NC group (*P* = 0.0009; Figure 4(a)), and the rate of wound healing in the miRNA-140 inhibitor group was higher than that in the inhibitor NC group (*P* < 0.0001; Figure 4(b)). Transwell assays (without or with Matrigel-coated membrane) identified less active migration and invasion in the miRNA-140 mimic group than in the mimic NC group (*P* = 0.0002 and *P* < 0.0001, respectively; Figures 4(c), and 4(e)). Compared with the inhibitor NC group, more active migration and invasion were observed in the miRNA-140 inhibitor group (*P* = 0.0034 and *P* < 0.0001, respectively; Figures 4(d) and 4(f)). The results indicated that miRNA-140 played an important role in inhibiting HCC cell migration and invasion.

### 3.7. Effect of miRNA-140 on the Cell Cycle of SMMC-7721

**Figure 5:**
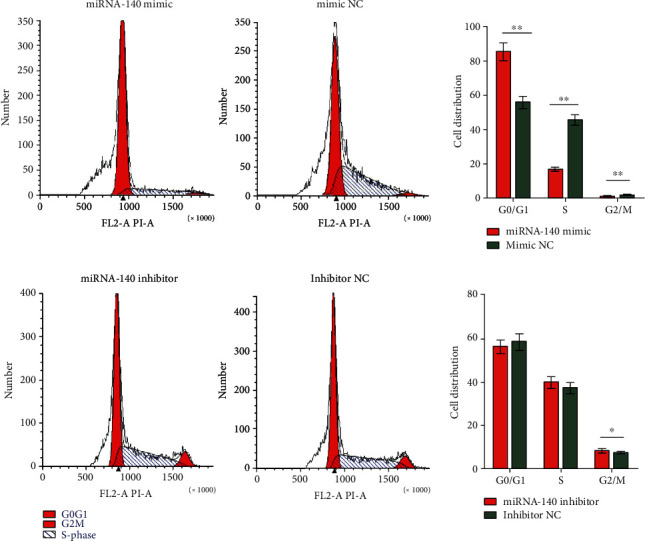
Effect of miRNA-140 on the cell cycle of SMMC-7721 cells.

We used flow cytometry to detect the cell cycle of SMMC-7721 cells 48 h after transfection to verify the effect of miRNA-140 on the cell cycle of SMMC-7721 cells. The percentages of cells in G0/G1, G2/M, and S phases were compared. A comparison of the percentages of cells in the three phases indicated that the miRNA-140 mimic and mimic NC groups had more cells in the G0/G1 phase (miRNA-140 mimic, 85.036 ± 5.022; mimic NC, 55.692 ± 3.289; *P* = 0.001), fewer cells in the G2/M phase (miRNA-140 mimic, 1.236 ± 0.073; mimic NC, 1.854 ± 0.109; *P* = 0.001), and fewer cells in the S phase (miRNA-140 mimic, 16.727 ± 0.988; mimic NC, 45.454 ± 2.684; *P* < 0.001) compared with the control group. The miRNA-140 inhibitor and inhibitor NC groups had fewer cells in the G0/G1 phase (miRNA-140 inhibitor, 56.112 ± 2.672; inhibitor NC, 58.195 ± 3.437; *P* = 0.454), more cells in the G2/M phase (miRNA-140 inhibitor, 8.250 ± 0.487; inhibitor NC, 7.350 ± 0.131; *P* = 0.037), and more cells in the S phase (miRNA-140 inhibitor, 39.706 ± 2.345; inhibitor NC, 37.121 ± 2.192; *P* = 0.236) than the control group. The results indicated that miRNA-140 overexpression blocked the G1 phase of the HCC cell cycle and inhibited the HCC cell cycle from the G0/G1 phase to the S phase. Therefore, the overexpression of miRNA-140 inhibits the proliferation of HCC cells (Figure 5).

### 3.8. miRNA-140 Gene Had No Effect on the Apoptosis of SMMC-7721 Cells

**Figure 6:**
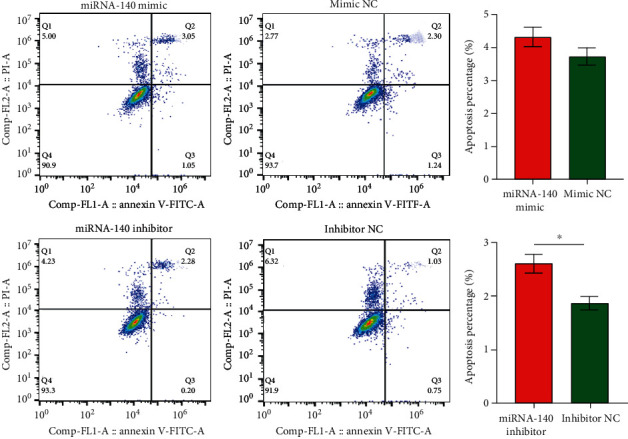
Effect of miRNA-140 on apoptosis of SMMC-7721 cells. ^∗^ represent *P* < 0.05.

We detected the effect of the upregulation and downregulation of miRNA-140 on SMMC-7721 cell apoptosis by flow cytometry to detect whether miRNA-140 affects apoptosis. The results showed no significant difference between the miRNA-140 mimic group and the mimic NC group (*P* = 0.1621). Although the inhibitor group and inhibitor NC group had a significant difference (*P* = 0.0405), this result was not enough to show that miRNA-140 has an effect on apoptosis. Therefore, we speculate that the miRNA-140 gene may not play an important role in the apoptosis of HCC cells (Figure 6).

### 3.9. Target Gene, GO Annotation, and KEGG Pathway Enrichment Analyses

**Figure 7:**
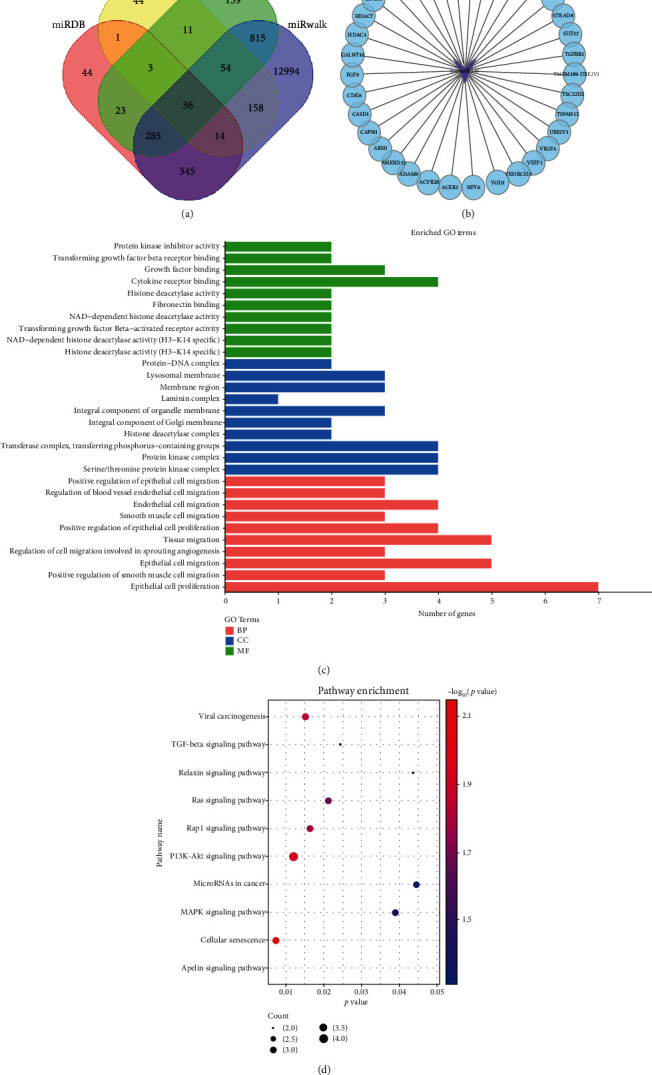
Predictive target gene and GO annotation and KEGG pathway enrichment analysis. (a) The Wayne diagram of the intersection of predictive genes. Pink is the predictive gene set of the miRDB database, yellow is the predictive gene set of the miRTarBase database, green is the predictive gene set of the miRWalk database, and purple is the predictive gene set of the TargetScan database. (b) The interaction network between miRNA-140 and mRNA; the purple triangle shows miRNA-140, and the blue dot shows the mRNA interacting with miRNA-140. (c) The GO function annotation of 36 target genes interacting with each other. Green represents a molecular function (MF), blue represents cell component (CC), and red represents the biological process (BP). (d) The enrichment analysis of KEGG (Kyoto Encyclopedia of Genes and Genomes) pathway of 36 target genes interacting with each other.

We predicted 751, 322, 14,701, and 1,386 miRNA-140 target genes through miRDB, miRTarbase, miRWalk, and TargetScan. Thirty-six genes were found in the intersection of the four databases (Figure 7(a)). A miRNA–mRNA regulatory network involved in the occurrence and development of HCC was established according to the predicted miRNA–mRNA pairs (Figure 7(b)). Next, we carried out GO annotation and KEGG pathway enrichment analysis for the 36 genes. We found that GO biological process analysis showed that the annotation of the miRNA-140 target genes was remarkably related to the positive regulation of smooth muscle cell migration, epithelial cell migration, cell migration involved in sprouting angiogenesis, tissue migration, smooth muscle cell migration, endothelial cell migration, regulation of blood vessel endothelial cell migration, and the positive regulation of epithelial cell migration (Figure 7(c)). In cell composition analysis, these genes were substantially enriched in protein kinase complex, histone deacetylase complex, laminin complex, and lysosomal membrane (Figure 7(c)). The molecular function analysis showed that these genes were enriched in histone deacetylase activity, NAD-dependent histone deacetylase activity (H3-K14 specific), fibronectin binding, and histone deacetylase activity (Figure 7(c)). In KEGG enrichment, the 36 genes were considerably enriched in cellular senescence, PI3K Akt signalling pathway, viral carcinogenesis, Rap1 signalling pathway, TGF-*β* signalling pathway, MAPK signalling pathway, relaxin signalling pathway, miRNAs in cancer, and apelin signalling pathway (Figure 7(d)).

## 4. Discussion

As one of the common malignant tumours, HCC can participate in the abnormal activation of signal pathways involved in gene mutations, chromosomal aberrations, abnormal secretion of growth factors, and epigenetic changes [21–23]. However, the effective treatment and early diagnosis of HCC still remain a worldwide medical issue [24, 25]. Relevant medical research and studies have predicted that the incidence rate of liver cancer will gradually decrease in the future with the improvement of medical quality and the popularisation and application of new technologies and therapies; however, liver cancer is still characterised by a high recurrence rate, short survival time, and poor prognosis [26]. Therefore, finding markers for the early diagnosis of HCC remains the focus of current relevant research. miRNA has become a promising biomolecule for the diagnosis, prognosis, and treatment of tumours with the development of miRNA research [27, 28].

The anticancer effect of miRNA-140 is reflected in a variety of tumour cells and tissues and affects the occurrence and development of tumours. Iorio et al. discovered that the expression of miRNA-140 decreases in ovarian cancer and its related cell lines by prediction analysis of microarrays [29]. miRNA-140-5p inhibits the progression of colorectal cancer by targeting vascular endothelial growth factor A and mediates the inhibition of Smad2 and autophagy to inhibit the survival and invasion of colorectal cancer stem cells [30, 31]. miRNA-140 has an inhibitory effect on the growth and metastasis of non-small-cell lung cancer cells, and its target is insulin-like growth factor 1 receptor [32]. In addition, oestrogen receptor *α* signalling pathway can downregulate the expression of miRNA-140, and the target of miRNA-140 to regulate the initiation of breast cancer cells is the transcription factor Sox2 [33]. Furthermore, miRNA-140 inhibits the invasion and migration of gastric cancer cells by downregulating HDAC4 [34]. miRNA-140 promotes the invasion and metastasis of oesophageal cancer cells by targeting slug genes to induce epithelial-stromal transformation [35]. Our experiments explored the expression of miRNA-140 in tumour tissues and adjacent normal tissues of patients with HCC. The results showed that the expression level of miRNA-140 was high in tumour tissues and low in adjacent normal tissues. In addition, the statistical results suggest that the elevated expression of miRNA-140 will increase the OS and DFS of patients. The expression of miRNA-140 is an independent protective factor for OS and DFS in patients with HCC. Thus, miRNA-140 can be viewed as a prognostic biological marker of HCC.

A variety of existing studies have confirmed the correlation between miRNA-140-5p and the metastases of ovarian cancer [36], colorectal cancer [31], lung cancer [37], and tongue cancer [38]. Takata et al. [39] confirmed that miRNA-140-5p has an inhibitory effect on HCC and affects the development of HCC, the expression of miRNA-140-5p decreases in hepatoma tissues and many hepatoma cell lines, and miRNA-140-5p regulates the signal pathways of TGF-*β* and MAPK by regulating the downstream target gene TGF-*β* receptor 1 and FGF9 and thus affects the transformation and proliferation of hepatoma cells. Yang et al. [19] discovered that miRNA-140-5p could act on the downstream target gene prolinyl isomerase Pin1, reduce the phosphorylation of protein kinase B and extracellular regulatory protein kinase, and thus block the activation of a variety of cancer-promoting signals. miRNA is involved in cell regulation, including cell metabolism, cell proliferation, cell apoptosis, differentiation, and cell response, after virus infection [40]. Cai et al. [41] demonstrated that sorafenib and AuNP–anti-miRNA-221 combination treatment exerts an anticancer effect on HCC via inhibiting miRNA-221/p27/DNMT1 signalling pathway. Lu et al. [42] uncovered the interaction between FLJ33360 and miRNA-140. miRNA-140 was downregulated in HCC tissues. Moreover, the knockdown of miRNA-140 could stimulate metastatic ability in HCC. In the present study, the influence of the stable upregulated miRNA-140 expression in HCC cells on their related biological processes was identified. Cell proliferation assay and colony formation experiment signified that miRNA-140 overexpression inhibited the growth of HCC cells. In addition, the results of wound healing assay and transwell assay indicated that miRNA-140 overexpression inhibited the migration and invasion of HCC cells. The miRNA-140 gene may not play an important role in the apoptosis of HCC cells. We predicted 36 target genes of miRNA-140, such as *ACER2* and *ACVR2B*, by using the intersection method of the miRDB, miRTarBase, miRWalk, and TargetScan databases. In KEGG enrichment analysis, these 36 genes were remarkably enriched in cellular senescence, PI3K Akt signalling pathway, TGF-*β* signalling pathway, and MAPK signalling pathway. These results suggested the critical role played by miRNA-140 in HCC.

## 5. Conclusion

Our results suggested that miRNA-140 was significantly downregulated in HCC tissues and that a high level of miRNA-140 expression was critical to achieve the long-term survival of patients with HCC. miRNA-140 might inhibit HCC cell proliferation, migration, and invasion. We predicted the target genes and pathways of miRNA-140 through bioinformatics, but further research and studies are needed to better understand the specific types of targeted regulatory genes and their molecular mechanisms. miRNA-140 could be a potential prognostic biomarker and therapeutic target for HCC.

## Figures and Tables

**Table 1:** Association between miRNA-140 expression and clinicopathological features.

Variable	*N*	Low (*n* = 40)	High (*n* = 40)	*χ* ^2^	*P*
Biological sex					
Female	11	6	5	0.105	0.745
Male	69	34	35		
Age, years					
<60	62	30	32	0.287	0.592
≥60	18	10	8		
Drinking history					
No	63	31	32	0.075	0.785
Yes	17	9	8		
Family history					
No	71	36	35	0.125	0.723
Yes	9	4	5		
BMI					
≤25	68	34	34	0.000	1.000
>25	12	6	6		
AFP					
<400	38	18	20	0.201	0.654
≥400	42	22	20		
Liver cirrhosis					
No	18	7	11	1.147	0.284
Yes	62	33	29		
Number of tumor					
<3	63	30	33	0.672	0.412
≥3	17	10	7		
Metastasis					
No	70	34	36	0.457	0.499
Yes	10	6	4		
BCLC stage					
0/A	51	21	30	4.381	0.036∗
B/C	29	19	10		
PVTT					
No	70	35	34	0.105	0.745
Yes	10	5	6		

Abbreviations: BMI: body mass index; AFP: alpha-fetoprotein; PVTT: portal vein tumor thrombus; BCLC stage: Barcelona Clinic Liver Cancer stage.

**Table 2:** Cox regression analyses of factors predicting disease-free survival and overall survival of HCC.

Variable	DFS			OS		
	HR	95% CI	*P* value	HR	95% CI	*P* value
Biological sex (female/male)	0.424	0.181-0.996	0.049^∗^	0.383	0.156-0.936	0.035^∗^
Age, years (<60/≥60)	0.394	0.161-0.966	0.042^∗^	0.327	0.130-0.822	0.017^∗^
Drinking history (no/yes)	0.963	0.371-2.504	0.939	1.355	0.533-3.441	0.523
Family history (no/yes)	1.529	0.569-4.109	0.400	1.473	0.526-4.127	0.461
BMI (≤25/>25)	1.047	0.375-2.923	0.930	0.811	0.297-2.22	0.684
AFP, ng/mL (<400/≥400)	1.771	0.814-3.853	0.149	1.286	0.605-2.734	0.514
Liver cirrhosis (no/yes)	0.604	0.269-1.357	0.222	0.589	0.263-1.321	0.199
Number of tumor (<3/≥3)	2.224	1.019-4.856	0.045^∗^	1.795	0.798-4.033	0.157
Metastasis (no/yes)	1.392	0.525-3.688	0.506	1.607	0.612-4.22	0.336
BCLC stage (0, A/B, C)	1.344	0.600-3.010	0.472	1.928	0.851-4.37	0.116
PVTT (no/yes)	2.519	0.824-7.697	0.105	1.468	0.487-4.429	0.496
miRNA-140expression (low/high)	0.357	0.175-0.726	0.004^∗^	0.42	0.210-0.837	0.014^∗^

Abbreviations: BMI: body mass index; AFP: alpha-fetoprotein; PVTT: portal vein tumor thrombus; BCLC stage: Barcelona Clinic Liver Cancer stage.

## Data Availability

The datasets used and/or analyzed during the present study are available from the corresponding author on reasonable request.

## Abbreviations

HCC: Hepatocellular carcinoma
qRT-PCR: Quantitative real-time polymerase chain reaction
BMI: Body mass index
AFP: Alpha-fetoprotein
PVTT: Portal vein tumor thrombus
BCLC stage: Barcelona Clinic Liver Cancer stage
OD: Optical density
MTT: 3-(4,5-Dimethylthiazol-2-yl)-2,5-diphenyltetrazolium bromide
PBS: Phosphate-buffered saline
FBS: Fetal bovine serum
DMSO: Dimethyl sulfoxide
DMEM: Dulbecco's modified Eagle medium
OS: Overall survival
DFS: Disease-free survival
GO: Gene Ontology
KEGG: Kyoto Encyclopedia of Genes and Genomes
BP: Biological process
CC: Cell composition
MF: Molecular function.
